# Editorial: Training methodology: a multidimensional approach for team sport, volume II

**DOI:** 10.3389/fpsyg.2024.1416380

**Published:** 2024-05-21

**Authors:** Filipe Manuel Clemente, Ana Filipa Silva, Rodrigo Aquino, Daniel Castillo, Javier Raya-González, José Afonso, Hugo Sarmento, Gibson Moreira Praça, Luca Paolo Ardigò

**Affiliations:** ^1^Escola Superior Desporto e Lazer, Instituto Politécnico de Viana do Castelo, Rua Escola Industrial e Comercial de Nun'Álvares, Viana do Castelo, Portugal; ^2^Department of Biomechanics and Sport Engineering, Gdansk University of Physical Education and Sport, Gdańsk, Poland; ^3^Sport Physical Activity and Health Research and Innovation Center, Viana do Castelo, Portugal; ^4^Sport Lab, Department of Sports, Centre of Physical Education and Sports, Federal University of Espírito Santo, Vitória, Brazil; ^5^Faculty of Education, University of Valladolid, Soria, Spain; ^6^Faculty of Sport Sciences, University of Extremadura, Avenida de la Universidad S/N, Cáceres, Spain; ^7^Faculty of Sport, Centre of Research, Education, Innovation, and Intervention in Sport (CIFI2D), University of Porto, Porto, Portugal; ^8^Research Unit for Sport and Physical Activity (CIDAF), Faculty of Sport Sciences and Physical Education, University of Coimbra, Coimbra, Portugal; ^9^Department of Sports, Universidade Federal de Minas Gerais, Belo Horizonte, Brazil; ^10^Department of Teacher Education, NLA University College, Oslo, Norway

**Keywords:** sports training, training methodology, athletic performance, coaching, sports performance

## Introduction

In the pursuit of optimal athletic performance, a multitude of factors converge, each playing a crucial role in defining an athlete's capabilities. Central among these determinants is the methodology employed in training by coaches. The efficacy of training methodology pivots upon various dimensions, encompassing the intensity and distribution of training loads, the selection of appropriate training methods, and the comprehensive management of athletes' physical and mental wellbeing. Integral to this approach is the ongoing assessment and monitoring of fitness levels, overall readiness, and technical-tactical proficiency.

The range of training methodology is expansive, comprising numerous complex factors that collectively strive toward an unique aim: achieving peak performance. Despite the significance of the complex interplay between these factors, research often tends to focus on isolated elements, neglecting the synergistic relationships and interdisciplinary insights that could drive athletic development further.

Rather than emphasizing the dominance of singular factors, this Frontiers topic advocated for an integrative approach to research, fostering collaboration across various domains. By exploring the intersections of training methodology with disciplines such as sports psychology, nutrition, and other pertinent areas of expertise, a broader understanding of optimal strategies was aimed to be achieved.

The current Research Topic served as a platform that welcomed original research studies and reviews delving into various sports such as swimming, football, and yoga. Collectively, these contributions brought new and disruptive knowledge based on sports sciences and coaching practices. Among the six published articles, authors explored experimental and observational analytic studies covering diverse topics, including electrocardiographic analysis in sports athletes, chronic adaptations related to erythrocyte oxidative stress, cognitive adaptation to swimming, and findings regarding predictors for the retention and dismissal of coaches. Below, readers will find a brief summary of each article included in our Research Topic.

### Summary of the included articles

The cognitive response in sports plays a crucial role in determining competitive performance. In a study of Lai et al., the impact of the 1,500 m freestyle, the longest pool-based swimming event in the Olympic Games, was analyzed. Thirteen male university swimmers participated in the study, performing the 1,500 m freestyle at maximal speed. Cognitive function, assessed from the perspective of hemoglobin oxygenation difference (Hbdiff), as well as freestyle performance at maximal speed, were evaluated before and after the competition. Utilizing a combination of cognitive tests and functional near-infrared spectroscopy (fNIRS), the study examined changes in cognitive function and neurobiological activity. The research underscores the cognitive implications of exhaustive 1,500 m freestyle swimming, attributing the observed decline in cognitive function to both physical fatigue and altered brain activation patterns, particularly within the prefrontal cortex. These findings clarify the intricate relationship between physical exertion and cognitive performance, emphasizing the importance of considering both physiological and neurobiological factors in comprehending such effects.

Current multidimensional approaches to team sports training rely on data collected during training and competition to manage loads effectively. Tracking devices have gained significant attention within this framework. In a recent scoping review by Ferraz et al. the authors aimed to explore the utility of tracking systems for analyzing physical performance in team sports. Following the PRISMA statement, they reviewed 79 studies, noting a high methodological quality, indicating scientific progress in the field. Many of these studies are descriptive, focusing on characterizing or comparing drills, training sessions, and matches. Notably, most research utilizes video or GPS tracking technologies in outdoor sports like football and rugby, with fewer studies focusing on indoor sports such as basketball and futsal. This discrepancy underscores the necessity for investigations across various contexts, as kinematics and mechanical responses are likely context-dependent. In conclusion, the authors propose a model to improve the practical application of tracking systems. This model suggests using the characterization of match demands to guide training planning alongside predictive studies to evaluate the impact of different training regimes on match performance.

The aim of the study by Kurtoǧlu, Konar et al. was to elucidate the impact of core exercises on blood oxidative stress parameters in amputee football players (AF), a topic which has not been extensively explored despite the well-established benefits of aerobic exercise in this context. Employing an experimental methodology, a cohort of eleven elite AF players was recruited and randomly assigned to either a core exercise group (CEG) or a control group (CG). Over an 8-week period, blood samples were collected before and after the core exercise program to assess various oxidative stress parameters, encompassing both erythrocyte and serum markers. Analysis of the results revealed significant disparities in serum total oxidant status (sTOS) and oxidative stress index (sOSI) values between the baseline and 8th week pre-aerobic training load (ATL) within the CEG. Similarly, noteworthy differences were observed in sTOS and sOSI values between the corresponding time points in the CG. In summary, the findings suggest a favorable influence of core exercises on blood oxidative stress parameters in AF players, particularly in terms of reducing blood total oxidant levels.

Amputee football players have great options to suffer cardiovascular abnormalities due to the higher energy consumption during activities and some changes in cardiovascular system due to amputation. This could have effects in the electrocardiography of this population, thus, it is necessary to the compare the electrocardiographic (ECG) parameters of amputee football players (AF) with other typologies to obtain information which favor the process training of AF. In this sense, Kurtoǧlu, Kurtoǧlu, et al. asessed the ECG of AF with the ECG of football players without disability (FP) and the ECG of sedentary individuals without disability (SI). For this purpose, 32 participants were involved in this study (9 participants in the AF group, 11 participants in the FP group, and 12 participants in the SI group). ECG recordings were done in the supine position in a silent room, and P-wave amplitude, P-wave duration, PR interval, QRS duration, RR intervals, QT interval, QTc interval, ST segment, Tp-e duration, Tp-e/QT, and Tp-e/QTc ratios were calculated. Specifically, AF players the amputee players differed from non-disabled subjects in P-wave amplitude (increase), QTc (increase) and QRS duration (decrease). However, these parameters not reach a pathological level, not increaising the risk for any arrhythmic in the AF players.

One of the most common practices in professional football is the mid-season change of coach, mainly for sporting reasons in which the team's performance is declining. Through a logistic regression model, the physical effects of mid-season replacements of head coaches were checked, analyzing which external load variables can predict retention or dismissal (Sousa et al.). The data included six head coaches during three entire seasons in which several locomotive variables along 4 weeks (i.e., training sessions) and four matches before and after the replacement of these coaches were collected. The results showed that the teams of dismissed head coaches presented better locomotor performance during competition, however, the arrival of the new head coaches resulted in better values during training. In addition Sousa et al., found that only the meters per minute in game variable could predict the coaches' dismissal or retention with an odd ratio of 32.4%. On the contrary, the other eight locomotive variables analyzed do not have significant effects on the odds of the dismissal or retention of the coaches. The main conclusion is that the arrival of new coaches can change the dynamics and intensity of training, but in the weeks leading up to dismissal (i.e., 4 weeks), that intensity decreases.

Coaches and practitioners organize the sports training process to enhance performance, reduce injury risk, and ensure good wellness status throughout the season. Therefore, scientific bases on different therapy and training strategies contribute to supporting coaches and sports scientists in training planning. Bucea-Manea-Tonis and Paun investigated the perspectives of Romanian elite athletes and coaches regarding the feasibility of integrating yoga practice into training regimens for injury prevention and medical recovery purposes. The study involved 500 athletes, coaches, and medical personnel from three universities in Romania. An online survey was conducted to assess athletes' experiences with integrating yoga into pre/post-training routines and its positive effects on reducing posttraumatic stress disorder (PTSD). Romanian athletes utilize yoga both before and after competitions to enhance focus, balance, muscle, and joint elasticity, cultivate a winning mindset, regulate emotions and PTSD symptoms, visualize their competition performance, and envision themselves as winners. Additionally, the survey revealed that yoga is perceived as beneficial for cardiac rehabilitation, neuropathic pain, pulmonary disease, orthopedic conditions, and muscle strain. The findings support the idea that integrating yoga into training activities holds promise for positively impacting athlete performance and mitigating the adverse effects of competitions.

## Author contributions

FC: Conceptualization, Writing – original draft, Writing – review & editing. AS: Conceptualization, Writing – original draft, Writing – review & editing. RA: Writing – original draft, Writing – review & editing. DC: Writing – original draft, Writing – review & editing. JR-G: Writing – original draft, Writing – review & editing. JA: Writing – original draft, Writing – review & editing. HS: Writing – original draft, Writing – review & editing. GP: Writing – original draft, Writing – review & editing. LA: Writing – original draft, Writing – review & editing.

